# Racial disparities in liver cancer: Evidence for a role of environmental contaminants and the epigenome

**DOI:** 10.3389/fonc.2022.959852

**Published:** 2022-08-22

**Authors:** Adriana C. Vidal, Cynthia A. Moylan, Julius Wilder, Delores J. Grant, Susan K. Murphy, Cathrine Hoyo

**Affiliations:** ^1^ Department of Biological Sciences, Center for Human Health and the Environment, North Carolina State University, Raleigh, NC, United States; ^2^ Department of Medicine, Division of Gastroenterology and Hepatology, School of Medicine, Duke University, Durham, NC, United States; ^3^ Department of Biomedical and Biological Sciences, Julius L. Chambers Biomedical Biotechnology Research Institute, North Carolina Central University, Durham, NC, United States; ^4^ Department of Obstetrics and Gynecology, Division of Research, School of Medicine, Duke University, Durham, NC, United States

**Keywords:** liver cancer, race, epigenetic, contaminants, epigenome

## Abstract

Liver cancer incidence has tripled since the early 1980s, making this disease one of the fastest rising types of cancer and the third leading cause of cancer-related deaths worldwide. In the US, incidence varies by geographic location and race, with the highest incidence in the southwestern and southeastern states and among racial minorities such as Hispanic and Black individuals. Prognosis is also poorer among these populations. The observed ethnic disparities do not fully reflect differences in the prevalence of risk factors, e.g., for cirrhosis that may progress to liver cancer or from genetic predisposition. Likely substantial contributors to risk are environmental factors, including chemical and non-chemical stressors; yet, the paucity of mechanistic insights impedes prevention efforts. Here, we review the current literature and evaluate challenges to reducing liver cancer disparities. We also discuss the hypothesis that epigenetic mediators may provide biomarkers for early detection to support interventions that reduce disparities.

## Introduction

Primary liver cancer incidence has tripled since the early 1980s, with most cases (~75%) classified as hepatocellular carcinoma (HCC). Liver cancer is among the fastest increasing cancers, and is the third leading cause of cancer-related deaths worldwide and in the US (1). While the incidence increased until 2015, it appears to have plateaued among Asians/Pacific Islanders. Among non-Hispanic Blacks and American Indians/Alaska Natives, the incidence of HCC continues to increase (2).

In the US, over 40,000 primary liver cancer cases are diagnosed annually, but the incidence not only varies by race/ethnicity, but also by geographic location. The highest liver cancer incidence is in the Western and Southern US and among ethnic minorities. Data from 2005 to 2014 suggest that the US age-adjusted incidence rate was 6–7.7/100,000 overall; yet, in Non-Hispanic Black individuals the rate was 10–13/100,000 and in Hispanics was 13–17/100,000 during the same period (3–6). Moreover, two-year survival is approximately 50%, and prognosis is poorer in minority populations (7–10). While deaths related to other malignancies such as lung, breast, and colorectal cancer declined over 40% from 1990–2016, liver cancer mortality is rising among both men and women (11). Consequently, liver cancer is projected to surpass breast and colorectal cancer by 2030 to become the leading cause of cancer-related death in the US (12). The underlying causes of this rapid increase that disproportionately affects racial minorities are poorly understood.

**Figure 1:**
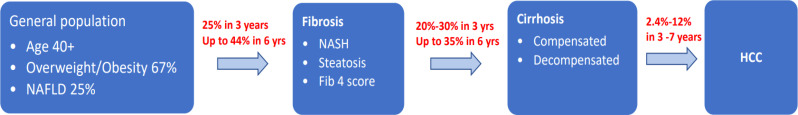
Conceptual model for progression from metabolic dysfunction, NAFLD, NASH, Cirrhosis and HCC.

The majority of HCC (over 90%) occurs in the background of chronic liver disease with cirrhosis of any etiology being the strongest risk factor (13). HCC has traditionally been driven by chronic liver disease from viral infections such as chronic hepatitis B virus (HBV) and hepatitis C virus (HCV). Increased vaccine rates and successful treatments have been associated with major declines in the incidence of HCC from these etiologies. However, the prevalence of these risk factors cannot fully explain the ethnic disparities observed. More recent data support a shifting of the underlying etiologies of HCC primarily due to the high prevalence of metabolic conditions that include obesity and diabetes, which increase the risk of non-alcoholic fatty liver disease (NAFLD) and its progression to NASH and cirrhosis. (14, 15). NAFLD represents a spectrum of chronic liver disease associated with obesity and insulin resistance that includes simple accumulation of fat in the liver (i.e., simple steatosis), to more severe non-alcoholic steatohepatitis (NASH) in which steatosis is complicated by necroinflammation and fibrosis, to cirrhosis, the end stage of fibrosis and scarring of the liver (16, 17). Although the potential for NAFLD to progress to fibrosis, cirrhosis and HCC is well established, progression, and thus HCC risk varies substantially by age and obesity status (**Figure 1**).

The prevalence of metabolic conditions such as obesity and type II diabetes (but not NAFLD) is also higher in Non-Hispanic Black, Hispanic, and Native American individuals. In the US, 31% of adults are overweight and approximately 10% are severely obese (18). National Health and Nutrition Examination Survey (NHANES) data from 2017–18 suggest that the age-adjusted prevalence of overweight/obesity varies by race among adults, with the highest incidence among Non-Hispanic Black (50%), followed by Hispanic (45%), Non-Hispanic white (42%), and Asian (17%) individuals. Similar race/ethnic prevalence patterns were reported for type II diabetes mellitus: among Non-Hispanic Black and Hispanic individuals, the prevalence is 13.2% and 12.8% respectively, whereas in Asian individuals it is 7.6% and in non-Hispanic white individuals it is 9% (19). Addressing disparities in liver cancer incidence and mortality requires rigorous investigation of upstream factors that give rise to metabolic derangement and progression to NAFLD, fibrosis, and cirrhosis that eventually leads to liver decompensation, liver cancer, and death. Advances in (epi)genomic sequencing technologies may help identify molecular mechanisms and events that promote liver deterioration. Molecular markers of liver cancer, that include genetic variants in genes such as PNPLA3, and epigenetic shifts largely identified from array data, are being developed into early detection tools aimed at reducing HCC risk and inherent ethnic disparities. Here, we review clinical and lifestyle risk factors for liver disease, the potential role of environmental exposures in liver cancer development, and the emerging role of epigenetics as a marker of past exposure to environmental contaminants, and contributor to liver cancer risk.

## Epidemiologic and lifestyle risk factors for liver disease

The Centers for Disease Control and the National Academy of Sciences estimates that environmental exposures account for at least 70% of variation in many chronic diseases risk, including liver diseases. Exposure to aflatoxin B_1_–lysine, cigarette smoking, mycotoxins, HBV and HCV infection, and poor access to treatment modalities increase liver disease risk including liver cancer. These findings now inform clinical practice to prevent these exposures and/or reduce the risk of progression. Conversely, habitual coffee intake and long-term statin and metformin use have protective effects on the liver. Indeed, much of this information is included in public health education. Association between comorbidities and drugs used to treat them, lifestyle factors such as diet, physical activity, and non-chemical stressors/social stressors and liver cancer are poorly characterized. Analgesics, such as acetaminophen, may also be linked to liver cancer. Together, data accumulated over the last two decades indicate that the prevalence of these risk factors disproportionately affects ethnic minorities, though data regarding risk factors among these populations remain sparse. Nonetheless, the prevalence of viral hepatitis infections, NAFLD, alcohol use, and exposure to mycotoxins, do not fully explain the continued HCC increase, especially the ethnic or geographic variation in liver disease.

Co-morbidities and the drugs used to treat them may also alter HCC risk. Prenatal acetaminophen exposure in mice results in loss of fetal liver stem cells, altering immune function (20, 21) and in adults, acetaminophen is the leading cause of acute liver injury/failure. Acetaminophen targets the liver and may interact with environmental contaminants such as cadmium that naturally target the liver, to increase risk of liver damage. Acetaminophen is used routinely by ~56% of the US population (22). Conversely, metformin and statins reduce HCC risk (23, 24). Accurate retrospective assessment of pharmaceuticals taken routinely for common ailments e.g., colds, or pain, is challenging. Another challenge is the lag in statistical methods development to investigate the effects of exposure to multiple drugs (i.e., drug mixtures).

Mounting evidence including high-quality randomized trials link anti-inflammatory diets, such a Mediterranean-style diet, to improvement in chronic diseases including cardiovascular diseases (25), reduced breast cancer incidence (26), and reduced metabolic diseases (27, 28). Coffee consumption is associated with lowered HCC risk while processed meat high in nitrates increases liver cancer risk and may also support liver cancer progression and mortality due to carcinogens released from nitrates that accelerate tumor growth. Diets rich in fruits, vegetables, and antioxidants reduce liver cancer risk, severity, and mortality (29–34). Certain dietary patterns (e.g., Mediterranean, glycemic index/load, or dietary inflammation index) decrease other biliary cancer risk (35–37), but little is known about the effects of diet on HCC prognosis. Moreover, non-pregnant minority adults report less adherence to these diets (38, 39). Mechanistically, anti-inflammatory diets reduce systemic free radicals and oxidative stress, leading to decreased circulating pro-inflammatory cytokines and chemokines (40–43).

Physical activity may reduce liver cancer risk and severity and improve outcomes in human studies and animal models. Inconsistent evidence supports the association between light, moderate, or vigorous physical activity and low liver cancer risk (44–47). Lack of consistency in findings is likely because physical activity tends to benefit subsets of populations, likely with other risk factors, such as smokers or obese individuals. However, a recent meta-analysis found that physical activity helped reduce liver cancer risk and mortality in a dose-dependent manner. At a minimum, two hours/week of physical activity was associated with reduced risk of liver cancer mortality (48).

Cigarette smoking is a source of non-occupational exposure to multiple exogenous chemicals, including cadmium, a chemical with oncogenic potential, and alcohol is associated with alcoholic cirrhosis such that these lifestyle factors may either modify or directly interact to increase HCC risk. Conversely, meta-analyses using data accumulated over the last two decades suggest that coffee intake reduces HCC and other liver cancer risk (49–54). However, the mechanisms underlying these connections are unclear.

## Social stressors

Social stressors captured at the neighborhood level are persistent risk factors for disparities in a range of cancer outcomes (55). In the US, neighborhood ethnic composition is a strong predictor of hazardous toxicant exposure (56–58). Early data suggests that the racial distribution of the geographic cluster with the highest cadmium exposure is 2% white, 78% Black, and 14% Hispanic (59). Neighborhood disadvantage scores revealed that disadvantage is associated with elevated exposure to environmental contaminants such as cadmium, a probable carcinogen, in adults (59).

## Gender differences in HCC

HCC risk is higher in males and mortality varies by sex, as do competing risk factors, e.g., moderate/heavy alcohol intake while overweight status is more common in men and obesity is more common in women. Conversely, women have higher concentrations of contaminants in their bodies, such as cadmium, compared to men who experience similar exposure levels. This may result from higher gastrointestinal absorption of cadmium (60) in women or from competitive binding of cadmium to transporters that are typically bound by nutritive elements such as iron and selenium and may be depleted (60). Moreover, poor cadmium excretion leads to bioaccumulation and increased urinary cadmium with age.

## Toxic environmental chemicals and liver diseases

Chronic environmental contaminant exposure is understudied yet may contribute substantially to metabolic dysfunction, fatty liver, fibrosis, and HCC. Increased industrial applications of toxic metals such as cadmium, arsenic, and lead as well as per- and poly-fluoroalkyl substances (PFAS) (e.g., perfluoro-octanoic acid; PFOA, or perfluoro-octane sulfonic acid; PFOS) coupled with their slow degradation has increased these environmental pollutants in atmospheric, terrestrial, and aquatic systems. Their persistence in the environment provides a stable exogenous source for human exposure. Once in the body, slow excretion leads to bioaccumulation in primary organs of metabolism, including the liver (61), with a half-life in the body of 30–45 years for cadmium (59, 62) and up to 5 years for PFOA (63–65). Toxic metals such as cadmium and arsenic are classified as probable human carcinogens by the International Agency for Research on Cancer (66) and ranked in the top ten environmental chemicals of concern by the Agency for Toxic Substances and Disease Registry (ATSDR) (67), while PFOA is classified as a possible carcinogen. Whereas hepato-toxic effects of contaminants such as cadmium at high levels characteristic of occupational settings are well-documented (reviewed in 66), data are limited regarding exposure at levels experienced by the general population.

PFAS are widely used in food packaging, flame-retardants, scratch-resistant coating, fire-fighting foam, and metal plating. Notably, PFAS were identified as drinking water contaminants throughout the US, with roughly 6 million Americans drinking water that exceeds EPA guidelines for safe levels of PFOA and PFOS (64). When all PFAS are considered or more stringent guidelines are used, the estimate is much higher. Additionally, metal exposure is also widespread; arsenic is naturally present in some water supplies and cadmium is a constituent of tobacco smoke and is present in some commercial fertilizers (68–71) such that ingestion of dietary staples contributes to exposure. In the US, dietary cadmium intake is estimated at ~1 µg/day (72, 73). In pregnant women in Durham, NC, cadmium and PFOA were found to co-contaminate house dust that can be ingested or inhaled (59). Serum PFAS levels are also higher in non-Hispanic white and Hispanic than in non-Hispanic Black pregnant women (74, 75). Further, among all adults, rural African Americans have higher concentrations (76). In contrast, cadmium body burden is highest in African American and Hispanic individuals (62, 77, 78)—populations with a higher HCC incidence. The US National Toxicology Program has called for further research on the effects of these environmental chemicals on organ dysfunction, including liver cancers (79). Multiethnic cohort investigations are needed to determine the impact of toxic metals and PFAS exposure on liver fibrosis and HCC.

Data linking environmental contaminants to NAFLD or HCC are limited. Data from *in vitro* and *in vivo* models accumulated over the last decade support the hypothesis that exposure to PFAS or toxic metals such as cadmium and arsenic induces NAFLD/NASH and liver carcinogenesis (66). However, doses used to induce liver diseases in experimental settings were orders of magnitude higher than those experienced by the general population. The hypothesis that exposure to chemicals such as cadmium increases fatty liver risk and progression to fibrosis, cirrhosis, and HCC is supported by weak evidence in humans. These data include autopsy data that demonstrate that concentrations of both toxic metals such as cadmium, and PFAS such as PFOS and PFOA, are higher in the liver than other organs sites with increasing cancer incidence (e.g., pancreas, ovary) (80–82), indicating that the liver is a main repository for these organic and inorganic chemicals. These autopsy data are supported by murine models data that have demonstrated significantly higher liver fat fractions consistent with fatty liver disease and hepatic neoplastic lesions, found in mice exposed to cadmium at concentrations equivalent to non-occupational exposure (83). In human populations, consistent with geographic information systems (GIS) data (59), findings based on a representative sample of Americans (NHANES) (84) suggest that urinary cadmium—an established dosimeter for long-term exposure, is higher in African American and Hispanic than in white individuals, and is associated with overall liver cancer risk, mortality, and the HCC precursors, NAFLD and NASH. However, there was a limited number of African Americans in the study and the data are cross-sectional.

Although these findings support higher body burdens of at least one toxic metal individually contributing to HCC and precursors such as NAFLD, NASH, and cirrhosis, multiple challenges to defining the link between environmental exposures and liver cancer remain. First, HCC incidence requires a population-based case–control design, relying on cancer registries for case identification. However, cancer registry-based rapid case ascertainment systems for case identification are ill-suited for studying HCC, since most (80%) cases are diagnosed solely based on radiographic imaging. Thus, case-control studies that rely on rapid case ascertainment systems may be biased toward the ~20% of cases whose identification relies on biopsy tissue from transplant patients. Consequently, ethnic minorities at higher risk of liver diseases are likely under-represented. Further, advances in mass spectrometry (MS) (e.g., liquid or gas chromatography (LC/GC) for PFAS and inductively coupled plasma mass spectrometry (ICP-MS) for metals, human data from NHANES, and from our group support environmental co-occurrence of PFAS, such as PFOA and PFOS, and toxic metals, such cadmium, arsenic, and lead (85). These toxins also co-occur in the blood or urine of Americans (86–89). Interaction profiles from *in vitro* models of the ATSDR (67) also support that at least for toxic metals, the effects of toxic metals such as cadmium, lead, and arsenic are synergistic. Yet, statistical methods to identify chemical mixtures contributing to health outcomes are limited and may require large sample sizes. Studies that focus on surmounting these challenges will greatly benefit the field.

## Epigenetic marks as biomarkers for early detection

Perhaps one of the biggest challenges in investigating the role of environmental contaminants in liver disease and cancer risk, in general, is the need for retrospective exposure assessment and comparing exposure odds in individuals with and without cancer. Indeed, case–control design is most efficient and is sufficient to investigate exposures such as urinary cadmium, an established dosimeter estimating the cumulative body burden over the life course, to investigate liver cancer etiology. However, the body burden of contaminants such as lead, arsenic, PFOA, and PFOS measured at cancer diagnosis are unlikely to reflect the body before diagnosis. This temporal ambiguity between environmental contaminant assessment and HCC is one of the main complications for causal inference. One way of circumventing this challenge requires molecular profiling that mediates exposure and outcomes.

While twin and familial studies estimate cancer heritability and its precursors such as obesity from 40 to 70%, cancer etiology is complex. Genetic loci contribute to less than 10% of obesity variation. Rather, heritable environmentally induced-epigenetic adaptation, including dysregulation of growth regulating genes, drives heritability, although the regions of the epigenome that contribute to liver diseases are undefined. Epigenetic marks act as exposure archives that approximate past exposure (90, 91). This is in part because epigenetic regulation, a means by which gene expression is altered in response to environmental exposures, can cause long-term changes in expression in mechanistic pathways contributing to liver injury, dysmetabolism, nutrient acquisition, fat deposition, appetite, and satiety. Both covalent DNA methylation at cytosines of CpG dinucleotides and histone modifications regulate chromatin structure and gene expression. The value of DNA methylation as an assay target is its stability. This enables its measurement from nearly any sample type, regardless of handling, by utilizing both targeted and high-throughput bisulfite sequencing methods.

## Future research direction using epigenetics

Human epigenetic data linking liver cancer and its precursors to epigenetic dysregulation has three main challenges that hamper identification of epigenomic regions mechanistically involved in cancer development. First, clinically accessible peripheral cells (e.g., blood or buccal cells) may not be appropriate surrogates for tissue types of etiologic significance to liver cancer. Second, epigenetic marks respond to environmental cues throughout the life course such that without serial samples, inference of cause-and-effect between obesity and any epigenetic alterations is difficult. Additionally, epigenetic marks associated with obesity are often identified from known regions or genes, targeted by function. Moreover, agnostic approaches use array technology (e.g., Golden Gate, 14K, 27K, 450K, or EPIC), but there are physical limitations such as the limited number of CpGs per array, and these approaches are selected based on predetermined criteria of likely significance. For example, while target regions have been selected to cover gene promoters and bodies, as well as CpG islands, with >28 million CpG sites in the genome, less than 5% are covered. Thus, the scope of affected regions is unknown. Another genome-wide tool, meDIP, covers ~40% of the genome, but is dependent on antibody precipitation of methylcytosine, and is thus more effective in CG-rich regions. Also, because meDIP captures only methylated sites, accurate quantitation of methylation percentage is not feasible. Reduced representation bisulfite sequencing is genome-wide but covers ~10% of CpG sites due to technological dependence on endonuclease recognition of specific sites. While these methods are all highly informative for measurable regions, many epigenetic regions occur at long (>10-20kb) distances from gene bodies, and in areas of low CG content. Thus, the coverage has selection/sequence bias.

Addressing these challenges in epidemiologic settings requires multiple approaches to identify epigenomic regions of functional relevance that link environmental exposures and liver dysfunction. These include using agnostic genome scale approaches such as whole-genome bisulfite sequencing or agnostic arrays (e.g., EPIC850 methylation array) and case and control specimens to identify genomic regions that differ between cases and controls in a cell type accessible for both cases and controls, e.g., blood. This step is followed by determination of the relevance of the marker, in affected cancer tissues. Among regions with a high likelihood of being functionally important, follow-up investigation determining if the epigenetic marks with case–control differences that are also found in relevant tissues are stable over time, and thus unlikely to be caused by disease, is performed to establish cause-and-effect. Finally, the biological significance in cancer is determined. Because these approaches have limitations to identifying epigenetic markers for liver cancer risk overtime, circumventing these challenges requires inclusion of molecular profiling that mediates exposures and outcomes in large cohorts with long-term follow up, such us the *All of Us* study (https://allofus.nih.gov/news-events-and-media/announcements/all-us-research-program-initial-protocol).

Another limitation of epigenetics studies is that environmental exposures affecting the epigenome may cause temporary changes in methylation that could be reverted after the exposure is no longer present. Thus, it is important to focus on CpG methylation marks that are stochastically established before specifications that control metastable epiallele expression (92) and imprinting control regions (ICR) that regulate imprinted gene monoallelic expression (93, 94). Methylation marker stability with age also makes them long-term ‘records’ of early exposures that are difficult to obtain through questionnaires or other exposure assessment assays (93). CpG methylation of metastable epialleles and ICRs is established before gastrulation and are mitotically heritable. Thus, epigenetic marks are similar across tissues and cell types throughout the individual’s life. Unlike metastable epialleles, however, ICRs are defined by parent-of-origin specific methylation marks that are important gene dosage regulators based on the allele’s parental origin. Consequently, in contrast to epigenetic marks controlling metastable epiallele expression, methylation marks regulating imprinted genes are similar across individuals (95, 96). Importantly, changes in ICR methylation patterns are implicated in adult-onset diseases suspected to have fetal origins, including neurological disorders, cancers, and metabolic diseases stemming from abnormal growth and nutrient acquisition disorders (97, 98). With the recent publication mapping the complete repertoire of human ICRs (99), examining the effects ICR dysregulation on liver diseases, including cancer, should yield new insights.

Chronic exposure to environmental contaminants characteristic of non-occupational settings results in subtle molecular adaptive responses detectable as methylation marks at epigenetically labile CG dinucleotides (100–102). Targeted methylation sequencing approaches demonstrated that cadmium alone or in a mixture with arsenic is associated with hypermethylation of the *DLK1/MEG3* imprinted domain in leukocyte-derived DNA (103). Conversely, untargeted whole genome bisulfite sequencing revealed that cadmium exposure was associated with differential methylation, at ~2,000 loci (104). Recent studies using Illumina Beadchip arrays also support that DNA methylation of two CG dinucleotides, measured in cell-free DNA, can distinguish HCC from cirrhosis with both sensitivity and specificity in excess of 90% (105). Intriguingly, these CG dinucleotides map to two genes that are key components of the extracellular matrix, epithelial to mesenchymal transition, and signaling (106). This is consistent with dying hepatocytes contributing to the pool of circulating DNA in plasma (107) and the observation that up to 70% of cell free DNA in HCC cases is contributed by the liver (107–109). While these data suggest methods for etiologic investigations and early detection using accessible cells of relevance circulating in plasma, the role of environmental contaminants in methylation alterations has not been examined. Further, racial minorities have not been included in case–control design, hampering causal inference.

Similarly, circulating cell-free RNA is comprised of different classes of RNA, including messenger, micro, circular, long-coding, transfer, ribosomal, and mitochondrial RNAs. RNA pools reflect physiological and pathophysiological insight into human health and have the potential for diagnostic and prognostic markers of disease and monitoring (110, 111). The most studied group of cell free RNAs are miRNAs that can target and regulate genomic output through multiple mechanisms (112) and are an emerging class of effector molecules regulated by diet (113). These miRNAs circulate throughout the body and due to their size and stability are found in most bodily fluids including blood, urine, saliva, breast milk, and tears (114, 115). The epigenome regulates miRNAs and in turn, miRNAs reciprocally regulate DNA methylation by inhibiting DNA-modifying enzymes (116). However, inclusion of racial minorities under-represented in epidemiological research studies remains low, which challenges the interpretation of existing data. Better representation of these groups is needed to understand disparities in liver cancer and in the development of early detection methods.

## Summary

Racial/ethnic disparities in the incidence of cancers such as HCC is paralleled by increased prevalence of environmental contaminants. The inflammatory effects of environmental exposures may be modulated by disparities in lifestyle factors, comorbidities, and higher body burden of environmental contaminants. Resilience factors such as anti-inflammatory diets may mitigate exposure effects and are linked with a lower liver cancer prevalence among ethnic minorities. However, establishing the effects of risk factors in epidemiologic studies is complicated by retrospective exposure assessment. These shortcomings may be circumvented by a more detailed knowledge of epigenetic responses linking environmental exposures to cancer outcomes.

## Author contributions

All authors listed have made a substantial, direct, and intellectual contribution to the work and approved it for publication.

## Funding

Funded by NIH R01HD098857, R01ES093351, and R01MD011746-S1.

## Conflict of interest

The authors declare that the research was conducted in the absence of any commercial or financial relationships that could be construed as a potential conflict of interest.

## Publisher’s note

All claims expressed in this article are solely those of the authors and do not necessarily represent those of their affiliated organizations, or those of the publisher, the editors and the reviewers. Any product that may be evaluated in this article, or claim that may be made by its manufacturer, is not guaranteed or endorsed by the publisher.
